# EEG assessment of brain dysfunction for patients with chronic primary pain and depression under auditory oddball task

**DOI:** 10.3389/fnins.2023.1133834

**Published:** 2023-03-24

**Authors:** Yunzhe Li, Banghua Yang, Zuowei Wang, Ruyan Huang, Xi Lu, Xiaoying Bi, Shu Zhou

**Affiliations:** ^1^School of Medicine, School of Mechatronic Engineering and Automation, Research Center of Brain Computer Engineering, Shanghai University, Shanghai, China; ^2^Shanghai Shaonao Sensing Technology Ltd., Shanghai, China; ^3^Division of Mood Disorders, Shanghai Hongkou Mental Health Center, Shanghai, China; ^4^Department of Neurology, Shanghai Changhai Hospital, Second Military Medical University, Shanghai, China

**Keywords:** chronic primary pain (CPP), depressive disorder, EEG, phase lag index (PLI), brain networks, convolutional neural network (CNN)

## Abstract

In 2019, the International Classification of Diseases 11th Revision International Classification of Diseases (ICD-11) put forward a new concept of “chronic primary pain” (CPP), a kind of chronic pain characterized by severe functional disability and emotional distress, which is a medical problem that deserves great attention. Although CPP is closely related to depressive disorder, its potential neural characteristics are still unclear. This paper collected EEG data from 67 subjects (23 healthy subjects, 22 patients with depression, and 22 patients with CPP) under the auditory oddball paradigm, systematically analyzed the brain network connection matrix and graph theory characteristic indicators, and classified the EEG and PLI matrices of three groups of people by frequency band based on deep learning. The results showed significant differences in brain network connectivity between CPP patients and depressive patients. Specifically, the connectivity within the frontoparietal network of the Theta band in CPP patients is significantly enhanced. The CNN classification model of EEG is better than that of PLI, with the highest accuracy of 85.01% in Gamma band in former and 79.64% in Theta band in later. We propose hyperexcitability in attentional control in CPP patients and provide a novel method for objective assessment of chronic primary pain.

## 1. Introduction

Chronic pain is defined as pain that lasts or recurs for more than 3 months, characterized by the interaction of biological, psychological, and social factors (Treede et al., 2015). At present, the etiology and pathogenesis of some chronic pain are not clear. The terms “somatoform pain disorders” or “functional pain syndromes” are usually used to describe this kind of pain. Such patients often go to various specialties of the hospital for repeatedly checking to legitimize their suffering, consuming a lot of medical resources. However, due to diagnostic uncertainty patients often feel guilty or angry and poorly understood. They are dissatisfied with the curative effect, and even have a crisis of trust in doctors (Serbic et al., 2022). In 2019, the IASP expert group participated in the revision and release of the International Classification of Diseases 11th Revision (ICD-11), which proposed the concept of “chronic primary pain (CPP)” (Nicholas et al., 2019), advocating that it be incorporated as a disease in its own right rather than a symptom (Treede et al., 2019). This diagnosis needs to be considered and targeted when the cause of chronic pain is not clear but is accompanied by significant emotional abnormalities and functional impairment.

Although there are still some disputes on the concept of CPP, more and more studies suggest that CPP may lead to specific changes in brain plasticity, which could be expected to be an objective diagnostic indicator of CPP. The recent review found that the gray matter volume of the cingulate cortex and insula of CPP patients decrease significantly while the right striatum gray matter increases (Wang et al., 2022). The duration of pain symptoms is positively correlated with the right brain volume and negatively correlated with the volume of the right anterior cingulate cortex and the right middle frontal gyrus gray atter. At the same time, CPP induces dysfunction of descending pain modulation, which is closely related to the serotonin system (Tao et al., 2019).

Clinically, patients with chronic pain have a higher risk of having depressive symptoms (Yalcin and Barrot, 2014). This comorbidity may indicate overlapped underlying neural mechanisms in chronic pain and depressive disorders (DD). As un particular type of chronic pain characterized by severe functional disability and emotional distress, CPP may present closer relationship with DD. However, there is still a lack of mechanistic studies exploring the similarities and differences between the two, which could provide the clinician with important diagnosis and treatment tools (Burke et al., 2015; Harth and Nielson, 2019).

Compared with other brain imaging technologies, EEG has a time resolution of milliseconds, which can measure the changes in brain neurophysiology (Boloukian and Safi-Esfahani, 2020; Yasin et al., 2021). On one hand, there have been many studies on depression-related EEG markers. Some researchers mainly use the degree of prefrontal lateralization of normal people and patients with depression to achieve classification (Jesulola et al., 2015; Palmiero and Piccardi, 2017), and propose that EEG characteristics can predict the heterogeneity of individual antidepressant drug responses to a certain extent. It is mainly found that the activities of Theta and Alpha frequency bands in the prefrontal and parietal lobes are related to depression (Pigoni et al., 2019). The latest research is based on the resting state EEG model optimized by computer. It is found that the neural activity of the prefrontal lobe can predict the treatment response of patients with depression to antidepressants (Wu et al., 2020). On the other hand, important efforts have been made in EEG biomarkers for chronic pain, although there is still disagreement on specificity and replicability (Mouraux and Iannetti, 2018; Ploner and May, 2018). For example, resting-state Theta and Gamma synchrony in frontal areas (Ta Dinh et al., 2019) and greater frontoparietal connectivity of the alpha oscillations (Ye et al., 2019) have been involved in the pathophysiology of chronic pain. Using the tonic pain model, we and other researchers have demonstrated that prefrontal cortex-related functions (i.e., cognitive task performances) and brain activities measured by EEG are related to pain tolerance (Zhou et al., 2015a,b, 2020) and recovery (Rustamov et al., 2021). Therefore, the extraction of EEG features through machine learning and other methods can help in individualized diagnosis and therapeutic monitoring of CPP.

The auditory oddball task can study the characteristics of attention resource allocation, working memory and information updating (Isreal et al., 1980; Johnson, 1986; Polich, 1986; Murphy et al., 2014). The auditory P300 evoked by this paradigm has been well applied in psychiatric diseases, and is the main candidate electrophysiological biomarker of psychiatric diseases (Jeon and Polich, 2003). The functional connectivity of EEG signals, especially the analysis of complex brain networks, may be more important for exploring the mechanism of brain activity in a task state. It has been used to study psychological diseases such as Alzheimer’s disease and autism (Steinmann et al., 2018; Bosch-Bayard et al., 2020). For functional brain networks, researchers have proposed many coupling methods, such as correlation-based method, partial order correlation-based method, and sparse method (Bullmore and Sporns, 2012; Sporns, 2013; Li et al., 2015; van den Heuvel et al., 2018). According to the existing research, the Phase Lag Index (PLI) is insensitive to the brain volume effect and can eliminate all indirect causality. Therefore, it is an excellent method to obtain functional brain network connectivity since the direct causality among the brain network nodes can be more accurately evaluated (Stam et al., 2007). Graph theory analysis involves filtering and transforming the functional connection matrix into a graph, and can be applied to investigate its topological structure or connectome of complex brain network (Farahani et al., 2019). Using this method, a recent systematic review has found significant group differences between chronic pain patients and healthy controls on certain overall graphical measures rather than nodal levels (Lenoir et al., 2021). Convolutional neural network (CNN) was used in the field of image recognition at the early stage and achieved very good classification results in the field of biological signal analysis, as well as good results in the field of auxiliary diagnosis of mental diseases (Acharya et al., 2018; Yao et al., 2020), but it has not been applied in the analysis and auxiliary diagnosis of chronic pain yet.

Therefore, this study will use task-state (i.e., auditory oddball paradigm) EEG feature extraction, mainly applying CNN as a deep learning algorithm for classification and complex brain network analysis to identify the neurophysiological characteristics of CPP different from DD, and meanwhile to explore its characteristic neural representation.

## 2. Materials and methods

### 2.1. Procedure

**FIGURE 1:**
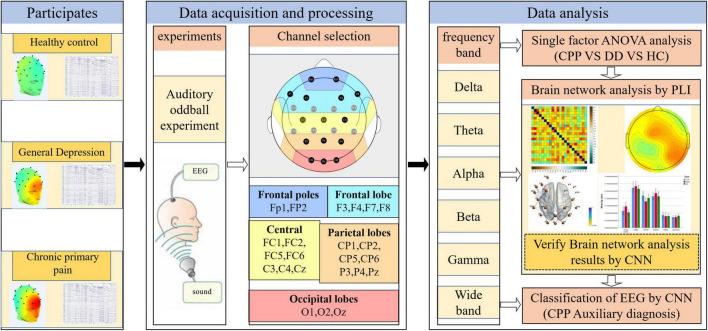
Flow chart of research methods. Collect EEG generated by auditory oddball stimulation in three groups of subjects, and then select 23 channels of interest for brain network analysis and CNN classification.

In this study, the auditory oddball task EEG data were collected from healthy subjects (HC) and patients with DD and CPP. First, using the PLI coupling to construct the brain functional connectivity network, the connectivity differences between groups of different brain regions in different frequency bands are compared and analyzed using graph theory and CNN. Then, the CNN algorithm is used to classify diseases based on EEG feature extraction in different frequency bands, providing a new method for objective evaluation of CPP. The processing flow of relevant data is shown in Figure 1.

### 2.2. Participates

In this study, twenty-two patients with CPP (13 females, 46 ± 9.62 years), twenty-two patients with DD (11 females, 46 ± 12.33 years), and twenty-three HCs (13 females, 49 ± 10.26 years) were recruited from the Shanghai Changhai Hospital, Shanghai Hongkou Mental Health Center and University of Shanghai, respectively, (Shanghai, China). All participants gave their written informed consent after the experimental procedure had been carefully explained. The research has been approved by the local research ethics committee.

Inclusion and exclusion criteria of subjects: chronic primary pain concerning the diagnostic criteria proposed by ICD-11: pain in one or more anatomical regions that (1) persists or recurs for longer than 3 months; (2) associated with significant emotional distress or functional disability (interference with activities of daily life and participation in social roles); (3) cannot be better accounted for by another chronic pain condition (American Psychiatric Association, 2013). Depressive disorder diagnosis was established according to the Diagnostic and Statistical Manual of Mental Disorders, 5th edition (DSM-V) criteria, as assessed by the structured clinical interview for DSM-V and Hamilton Depression Scale (HAMD). The two categories of patients also need to meet the following inclusion criteria: (1) Age 18–65 years old; (2) Junior high school degree or above; (3) Not taking medicine at the initial diagnosis or more than 6 months after drug withdrawal; (4) Volunteer to participate in this study and sign the informed consent form. Exclusion criteria: (1) Serious cognitive impairment or hearing disability, unable to cooperate in the completion of project-related assessment tests; (2) Serious physical diseases; (3) Severe psychiatric symptoms; (4) Abuse of psychoactive substances; (5) Suffering from diseases that can cause secondary chronic pain.

All subjects were instructed to fill in SDS for self-evaluation of depression level (Table 1). Two groups of patients were assessed by the physician with HAMD. The subjective and multidimensional experience of pain in CPP patients was quantitatively measured using the Short-Form McGill Pain Questionnaire (Dworkin et al., 2009). It comprises three subscales: a pain rating index (PRI) describing the qualities of pain, a 10 cm visual analog scale (VAS) describing the intensity of averaged daily pain during the past 2 weeks, and a present pain intensity (PPI) index describing the intensity of current pain.

**TABLE 1:** Number of subjects in three categories (N), average age, years of education, sex ratio, the average score of HAMD, the average score of SDS, and the average score of McGill Questionnaire.

		HC	DD	CPP
N		23	22	22
Average Age/Year		49 ± 10.26	46 ± 12.33	46 ± 9.62
Education/Year		10 ± 2.72	10 ± 4.73	10 ± 4.37
Male/Female		10/13	11/11	9/13
HAMD scale		5.46 ± 2.37	19.61 ± 4.01	20.83 ± 3.74
SDS scale		47.25 ± 3.55	69.28 ± 6.72	70.36 ± 5.68
McGill Questionnaire	PRI	0.98 ± 0.87	1.26 ± 0.65	12.47 ± 1.59
	VAS	1.21 ± 0.73	1.74 ± 0.67	6.42 ± 2.33
	PPI	0.20 ± 0.20	0.25 ± 0.24	3.73 ± 0.47

### 2.3. Experimental paradigm

**FIGURE 2:**
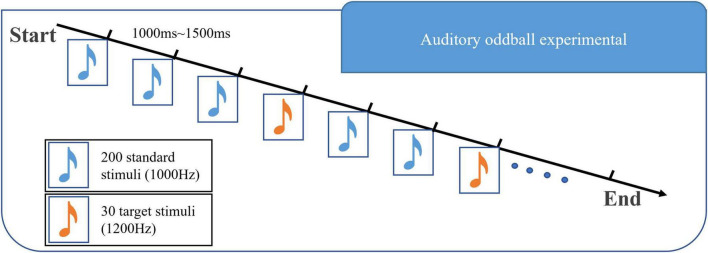
Diagram of the experimental paradigm in this paper. This experimental paradigm includes two kinds of stimuli: 1,000 Hz majority stimulus and 1,200 Hz target stimulus. The stimuli are arranged in a pseudo-random manner, with an interval of 1,000–1,500 ms between each stimulus.

Auditory oddball task was applied. The stimulus materials for the auditory oddball task were composed of 30 target stimuli (1,200 Hz, 75 dB, 50 ms) and 200 standard stimuli (1,000 Hz, 75 dB, 50 ms). The stimuli were arranged in a pseudo-random order with an interval of 1,000–1,500 ms between each stimulus. Before the experiment, there was a training part consisting of 10 tones, including two target stimuli. Subjects were asked to identify the tones with a low probability of occurrence, count them in silence, and then report to the researchers. The experimental program was written by E-Prime 1.0 software of Psychology Software Tools in the United States. The presentation mode of the stimulus sequence is shown in Figure 2.

### 2.4. Data acquisition and preprocessing

The experimental data acquisition equipment is 32 channels high-density EEG acquisition equipment made by Brain Productions Company in Germany. The recording software is based on the Vision Recorder system developed by the above company. During the whole experiment, the electrode was within the range below 30 k Ω, and the sampling rate was 1,000 Hz. The EEG cap was set with a 0.1–100 Hz band-pass filter in the default setting of the headset. To reduce the calculation cost during data processing, the data was down-sampled to 250 Hz. The impedance of recording electrodes was kept at less than 5 k Ω. The collected data were preprocessed using the EEGLAB toolbox (v2019.1) of MATLAB (R2020a) software: select 23 electrodes were placed on the scalp based on the international 10–20 electrode placement system and take Cz as the reference electrode. See Figure 1 for the selected electrodes and functional area division (Delorme and Makeig, 2004). Take the target stimulus as 0 times, select −0.2 s∼0.8 s data as a trial, and remove the components of eye and muscle electricity through independent component analysis.

### 2.5. Brain network construction and coupling

**FIGURE 3:**
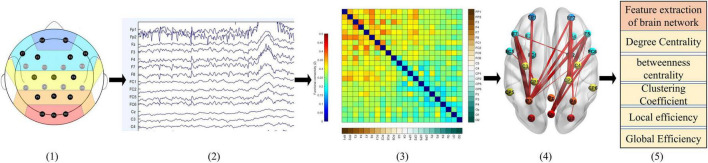
Typical construction process of functional brain network. From left to right : **(1)** the electrode of interest; **(2)** the preprocessed EEG; **(3)** the connection matrix calculated by PLI; **(4)** sparsely processed and mapped to the 3D human brain model; **(5)** carry out feature analysis on the sparse brain network.

As Figure 3 shows: (1) First, EEG data is collected and preprocessed; (2) To establish the functional connection, it is necessary to obtain the time series of brain activities in different regions; (3) According to these sequences, aggregation measurement is used to calculate the correlation of these sequences, so that the brain network is represented as a correlation matrix; (4) The consistency threshold method is applied to the matrix (30% of the connections are reserved) to retain the most significant part of the features so that the connection strength in different brain regions can be visually expressed; (5) Finally, the graph theory features are calculated and analyzed.

Before calculating PLI, the analytic signal based on the Hilbert transform is used to determine the instantaneous phase, to calculate the phase synchronization. The calculation formula of PLI is (Stam et al., 2007):


(1) $$P\,L\,I_{x\,y}\,\left(f\right)=\left|<s\,i\,g\,n\,\left(\phi_{x}\,\left(f\right)-\phi_{y}\,\left(f\right)\right)>\right|$$

Where < > represents the expected value, ∅_*x*_(*f*)−∅_*y*_(*f*) represents the phase synchronization of *x* and *y* channels/brain region signals at *f* frequency. The value range of PLI is [0,1], where 1 represents complete phase synchronization and 0 represents no phase synchronization.

### 2.6. Graph theory analysis of complex brain network

Graph theory is a method that can be applied to brain networks. It can describe the topological structure of complex networks and the changes in different network metrics in networks. The diagram is composed of a group of nodes (electrode array) and their connections (edges). For the quantification of graph topology, there are many measures. In this study, the following indicators were selected to conduct in-depth research on the constructed functional brain network.

#### 2.6.1. Node degree centrality (*DC*)

*DC* is the most direct metric to characterize node centrality in network analysis. The calculation formula for the degree centrality of a node is as follows (Boccaletti et al., 2006):


(2) $$D\,C_{i}=\frac{k_{i}}{N-1}$$

Where, *k_i_* represents the number of existing edges connected to node *i*, and *N-1* represents the number of edges connected to node *i* and other nodes.

#### 2.6.2. Node betweenness centrality (*BC*)

The intermediate number can reflect the importance of a node or edge in the network. It is the number of shortest paths through a node or edge in the network (Newman, 2005). It is defined as follows:


(3) $$B\,C\,\left(i\right)=\frac{2\,\sum_{h<j\in V,h\neq 1,j\neq 1}g\,h\,j\,\left(i\right)}{\left(N-1\right)\,\left(N-2\right)\,g\,h\,j}$$

Where, *g*_*hj*_ is the number of all shortest paths from node *h* ∈ *V* to node*j* ∈ *V*, *V* is the set of all nodes in the network, *g*_*hj*_(*i*) represents the number of all shortest paths from node *h* ∈ *V* to node *j* ∈ *V* passing through node *i*.

#### 2.6.3. Clustering coefficient (*CC*)

*CC* Of brain region node refers to the ratio of the number of edges connected by all nodes adjacent to the node to the maximum possible number of edges connected between these adjacent nodes. *CC* is often used to describe the degree of network integration or node density. The *CC* of node *i* represents the ratio of the current number of edges between all its neighboring nodes to the maximum number of edges that can exist between all its neighboring nodes. The larger the *CC* is, the closer the local connection of the node in the network is (Freeman, 1978):


(4) $$C\,C_{i}=\frac{2\,t_{i}}{k_{i}\,\left(k_{i}-1\right)}$$

*CC*_*i*_ is the clustering coefficient of node *i*, *t_i_* is the number of triangles formed by node *i*, *k_i_* is the degree of node *i*.

#### 2.6.4. Local efficiency (*E*_*loc*_)

*E*_*loc*_ measures how to efficiently spread information through the direct adjacent nodes of the node, measuring the local information transmission capacity of the network. The local efficiency of any node *i* is (Stam et al., 2007):


(5) $$E_{loc}(i)=\frac{1}{N_{G_{i}}(N_{G_{i}}-1)}\sum_{j\neq k\in G_{i}}\frac{1}{l_{j,k}}$$

*G_i_* refers to the subgraph formed by the neighbors of node *i*, *l*_*j,k*_ represents the shortest path length between nodes *j*,*k*.

#### 2.6.5. Global efficiency (**E**_**g***lob*_)

Global Efficiency is used to represent the degree of aggregation of nodes in the graph, and is defined as follows (Stam et al., 2007):


(6) $$E_{g\,l\,o\,b}=\frac{1}{N}\,\sum_{i=1}^{N}E_{i}=\frac{1}{N}\,\sum_{i=1}^{N}\frac{\sum_{j\in N,j\neq i}d_{i\,j}^{-1}}{N-1}$$

Where *E_i_* represents the efficiency of node *i*.

### 2.7. Deep learning algorithm for classification

This paper uses a compact CNN that can be used for various EEG signal classification tasks, including event-related potentials (ERP) (Prabhu et al., 2020). In addition, this article improves on the CNN and reduces the first AvgPool2d layer kernel size and stride by half, enabling CNN to classify the PLI matrix (22 × 22). Its architecture is shown in Table 2. The input of this model is raw EEG data, including channel number, and time sample. To limit the number of trainable parameters, the architecture adopts depth and separable convolution. The initial combination of 2D convolution and depth convolution allows each temporal filter to learn spatial filters (Schirrmeister et al., 2017). At the same time, the number of spatial filters learned from each feature map is controlled by the depth parameter. After each convolution, batch normalization is performed to achieve model stability. In addition, the dropout layer is used to significantly reduce overfitting (Mukhtar et al., 2021). The final multi-category classification layer uses the SoftMax function.

**TABLE 2:** The improved CNN network structure that classifies the PLI matrix, including the layer, the output shape of data, and the number of parameters.

Layer	Output shape	Param
Conv2d	[32, 8, 22, 22]	1,000
BatchNorm2d	[32, 8, 22, 22]	16
Conv2d With Constraint	[32, 16, 1,22]	352
BatchNorm2d	[32, 16, 1, 22]	32
ELU	[32, 16, 1, 22]	0
AvgPool2d	[32, 16, 1, 11]	0
Dropout	[32, 16, 1, 11]	0
Conv2d	[32, 16, 1, 12]	352
Conv2d	[32, 16, 1, 12]	256
BatchNorm2d	[32, 16, 1, 12]	32
ELU	[32, 16, 1, 12]	0
AvgPool2d	[32, 16, 1, 1]	0
Dropout	[32, 16, 1, 1]	0
Conv2d	[32, 3, 1, 1]	51
Log SoftMax	[32, 3, 1, 1]	0

### 2.8. Model training

**FIGURE 4:**
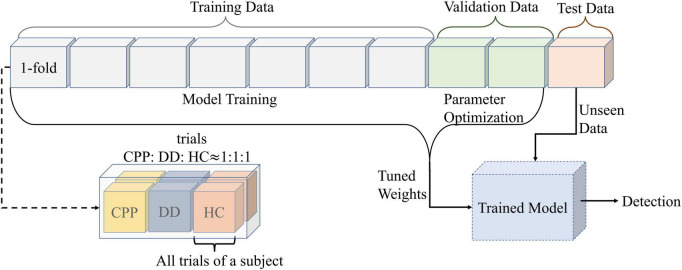
The data partition diagram used to create the model. Divide all data into 10-folds. The number of trials in each fold is the same, and the number of CPP, DD, and HC are uniform. At the same time, ensure that all data of each subject only exists in onefold. Divide the 10-folds into test set, validation set and training set according to 1:2:7. The training data is used for the training model, and the validation data is used for the parameter optimization. After adjusting the model weights for both sets of data together, the test set is used for prediction.

When using CNN for training, this study uses 10-fold cross-validation processing method, in which one random fold is used as the test set, the other twofolds are used as the validation set, and the rest as the training set. It also ensures that three types of subjects are evenly distributed in each fold and that the data of each subject only exists in the same fold. At the same time, each dataset (training set, validation set, and test set) is disordered before the training stage (Figure 4).

The network is trained using the backpropagation algorithm (Hung and Adeli, 1993) with a batch size (the number of training samples in iteration) of 32. An optimization algorithm, namely adaptive moment estimation (Adam) (Mukhtar et al., 2021) is adopted in this work to update the parameter of the proposed network structure. It was observed it enables the network to converge at a faster rate thereby improving the efficiency of the training process.

The following equation is used to update the 1st-moment estimate (Kingma and Ba, 2014). All operations on vectors are element by element.


(7) $$m_{t}=\beta_{1}\,m_{t-1}+\left(1-\beta_{1}\right)\,{\left[\frac{\partial c}{\partial \theta }\right]}_{t}$$

Where *m*, *t*, *c*, θ_*t*_ and $\frac{\partial c}{\partial \theta }$ are defined as the 1st-moment vector, timestep, cost function, resulting parameters (weights), and gradient, respectively. The parameters β_1_ and β_2_ represent the exponential decay rates which are chosen to be 0.9 and 0.999, respectively, (Kingma and Ba, 2014).

The following equation is used to update the 2nd-moment estimate (Kingma and Ba, 2014).


(8) $$v_{t}=\beta_{2}\,v_{t-1}+\left(1-\beta_{2}\right)\,{\left[\frac{\partial c}{\partial \theta }\right]}_{t}^{2}$$

The following equations are used to compute the 1st and 2nd-moment estimates, respectively, (Kingma and Ba, 2014).


(9) $${\hat{m}}_{t}=\frac{{\hat{m}}_{t}}{1-\beta_{1}^{t}}$$

(10) $${\hat{v}}_{t}=\frac{{\hat{m}}_{t}}{1-\beta_{2}^{t}}$$

In addition, the following equation is used to update the weights of links connecting the layers (Kingma and Ba, 2014).


(11) $$\theta_{t}=\left[1-\frac{\alpha \,\lambda \,\left(1-\beta_{1}\right)}{\sqrt{{\hat{\upsilon }}_{t}}+\epsilon }\right]\,\theta_{t-1}-\frac{\alpha \,{\hat{m}}_{t}}{\sqrt{{\hat{\upsilon }}_{t}}+\epsilon }$$

Where α and ϵ denote the learning rate and numerical value and are set at 1×10^−4^ and10^−8^, respectively, in this work. The variable λ, known as the regularization parameter, is also one of the essential parameters during training to prevent data overfitting. It is tuned to 0.2 in this work.

To avoid overfitting and improve the generalization, the dropout (Srivastava et al., 2014) technique is applied to the fully-connected layers 6 and 12. During training for each mini-batch, some of the neurons from these layers are selected randomly and dropped. This forces the model to learn from a subset of input features and not the entire input features. The value is set to 0.5, i.e., the probability of a neuron being retained during training is 50% and the probability of a neuron being rejected is 50%.

In this paper, the negative log-likelihood loss function is used to deal with multi-classification problems (de Boer et al., 2005). The input is the logarithmic probability value. For the batch data*D*(*x*,*y*) containing *N* samples, *x* is the output of the neural network and is normalized and logarithm zed. *y* is the category label corresponding to the sample, and each sample may be one of *C* categories.

*l_n_* is the loss corresponding to the *n*th sample, the value range is [0, C-1]:


(12) $$l_{n}=-w_{y_{n}}\,x_{n,y\,n}$$

*w* is used for sample imbalance between multiple categories:


(13) $$w_{c}=weight\left[c\right]\cdot 1\left\{c\neq ignore\_index\right\}$$

The default value of reduction is mean, and the corresponding *l*(*x*,*y*) is:


(14) $$1\,\left(x,y\right)=\sum_{n=1}^{N}\frac{1}{\sum_{n=1}^{N}w_{y_{n}}}\,l_{n}$$

### 2.9. Technology validation

F1 Score is an indicator used to measure the accuracy of the two-class model in statistics. It can be seen as a weighted average of the model’s accuracy and recall. Its value range is [0, 1]. The larger the value is, the better the model is.


(15) $$F\,1-s\,c\,o\,r\,e=\frac{2\,\left(r\,e\,c\,a\,l\,l*p\,r\,e\,c\,i\,s\,i\,o\,n\right)}{r\,e\,c\,a\,l\,l+p\,r\,e\,c\,i\,s\,i\,o\,n}$$

In multi-classification problems, Macro-F1 is usually used (Supratak et al., 2017).


(16) $$m\,a\,c\,r\,o-F\,1=\frac{1}{n}\,\sum_{i=1}^{n}F\,1-s\,c\,o\,r\,e_{i}$$

Where *n* is the number of categories and *i* is the number of categories.

### 2.10. Statistical analysis

SPSS software was used for statistical analysis. One-way analysis of variance analysis (ANOVA) was used for the comparative analysis of brain networks of three groups, where group category was used as a factor, and the average value of each channel after the superposition of the brain network connection matrix was used as a dependent variable. The Tukey method was used as a *post hoc* analysis for testing differences between groups.

## 3. Results

### 3.1. Functional connectivity

One-way ANOVA found that the brain networks of the three groups are statistically different except for the Beta band (Table 3). Further *post hoc* analysis based on the Tukey method (Table 4) shows that in the Gamma band there was difference in network characteristics between CPP and HC subjects but not between CPP and DD or DD and HC. In Delta and Alpha bands, a significant difference was present between CPP and DD, DD and HC, but not between CPP and HC. In the Theta frequency band only, the *P*-values between groups were all less than 0.05, indicating that different groups possess independent brain network characteristics in this frequency band.

**TABLE 3:** One-Way ANOVA analysis of the mean values of brain networks in three groups of people (95% confidence interval).

Frequency band	Sum of squares	Mean square	F	P
Wide band	0.044	0.22	57.617	< 0.0001
Delta	0.008	0.004	7.670	0.001
Theta	0.027	0.014	74.315	< 0.0001
Alpha	3.984	1.992	195.245	< 0.0001
Beta	< 0.0001	< 0.0001	1.560	0.218
Gamma	0.001	< 0.0001	7.662	0.001

**TABLE 4:** Significant *P*-Values for *post-hoc* comparisons based on Tukey’s method of ANOVA.

Frequency band	CPP vs. DD	CPP vs. HC	DD vs. HC
Wide band	0.995	< 0.0001	< 0.0001
Delta	0.025	0.500	0.001
Theta	< 0.0001	< 0.0001	< 0.0001
Alpha	< 0.0001	0.601	< 0.0001
Gamma	0.079	0.001	0.211

**FIGURE 5:**
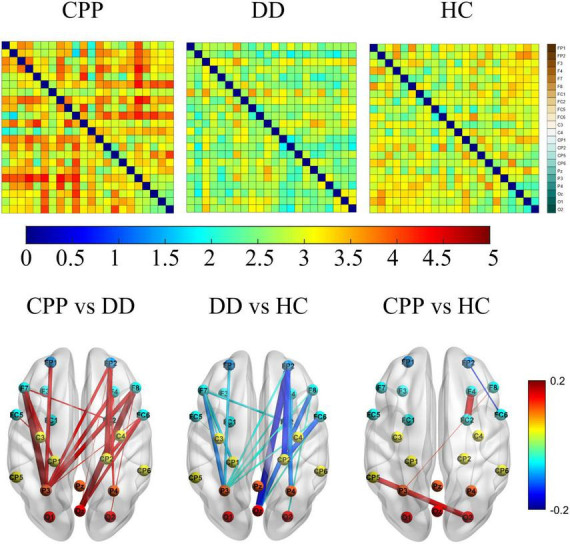
The above figure shows θ the functional connection matrix of band CPP, DD, and HC have a value range of [0, 5], and its electrode channels from top to bottom are FP1, FP2, F3, F4, F7, F8, FC1, FC2, FC5, FC6, C3, C4, CP1, CP2, CP5, CP6, Pz, P3, P4, Oz, O1, and O2. The following figure shows that the matrix values are subtracted (from left to right are CPP-DD, DD-HC, CPP-HC) and the most significant difference characteristics are retained by 30%, and its value range is [–0.2, 0.2].

To further explore the specific differences in brain networks among the three groups, we carried out a functional connectivity matrix analysis of CPP, DD, and HC and compared the differences after subtraction and the consistency threshold method. See Figure 5 for Theta band results (see Supplementary material for results in other bands). The results show that in the CPP group, the Theta frequency band connection between parieto-occipital electrodes (P3, O2) and prefrontal electrodes (FP1, FP2) is significantly enhanced (the connection strength of 4–5); in the HC group, the connections between electrode Cp5 and O2, electrode FC2 and F4 are slightly enhanced (the connection strength of 3–4); in DD group, the connection strength is the weakest (the connection strength of 1–3). Further difference comparison shows that the CPP group has significantly increased connectivity between the prefrontal area and the posterior parietal area compared with the DD group, while the difference between CPP and HC groups is mainly reflected in the stronger connectivity of CP5-O2 and F4-FC2; Compared to HC group, the connectivity strength of brain network in DD subjects is inhibited, mainly in the form of weakened connectivity between the parieto-occipital area and the prefrontal area.

### 3.2. Complex brain network

**FIGURE 6:**
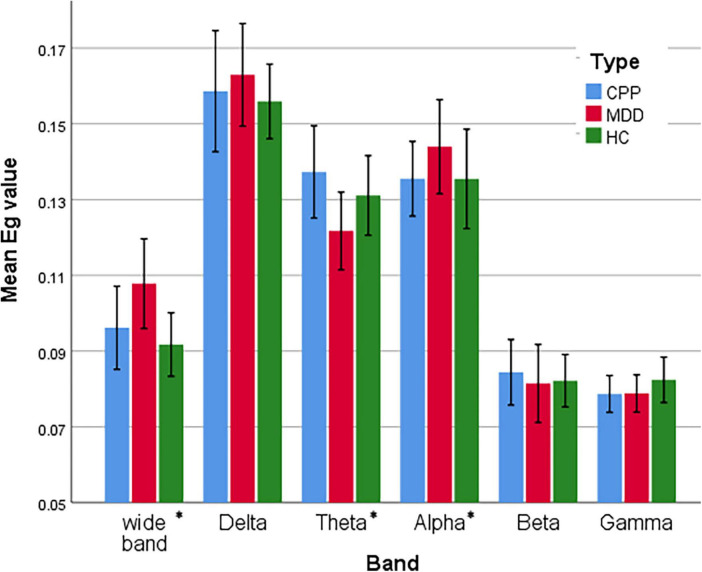
Statistical comparison chart of overall efficiency of three groups of people in six frequency bands. The error interval takes the value of 95%. *P < 0.05.

We further analyzed and compared the global feature revealed by *E*_*glob*_, as shown in Figure 6. The *E*_*glob*_ of DD subjects in Delta and Alpha bands is higher than the other two groups of subjects, while it is inhibited in the Theta band. This is also verified by the classification results of the PLI matrix using CNN (see Section “3.3. Classification accuracy of different frequency bands”). The difference between CPP and HC subjects is mainly reflected in the significant enhancement of the Theta band.

**FIGURE 7:**
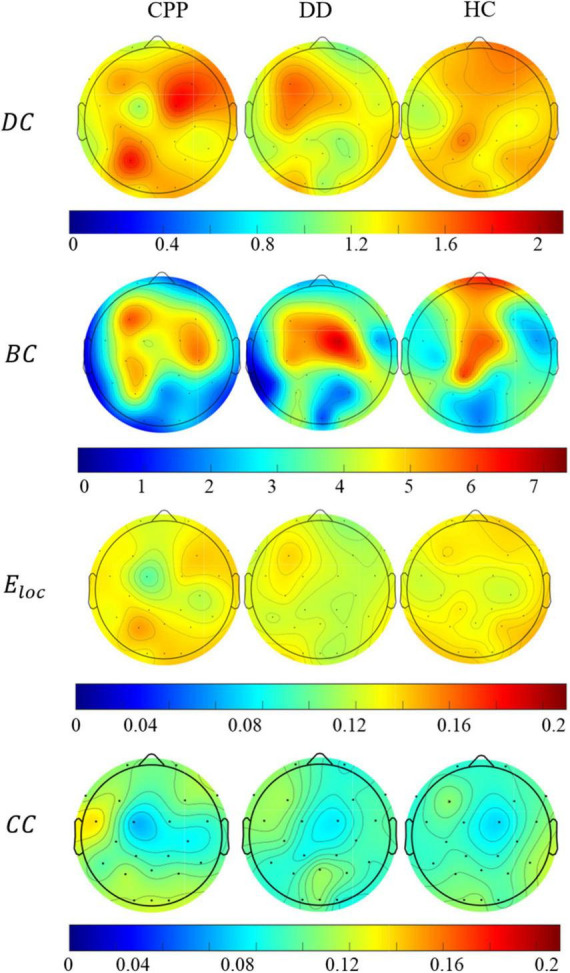
The average distribution of degree centrality, median centrality, local efficiency, and clustering coefficient of the three groups of people in Theta band.

At the node level, *DC*, *BC*, *E*_*loc*_, and *CC* of the three groups in the Theta band is also different, as shown in Figure 7. The *DC* intensity of CPP subjects is higher in the right frontal and left parieto-occipital region than in the other two groups. This result is consistent with the aforementioned trend of brain network connection intensity in the Theta band. In the distribution of *BC*, the intensity in the central area of CPP subjects is lower than that of the other two groups, and the intensity of *BC* in the prefrontal and temporal areas of CPP and DD is lower than that of HC subjects. Similarly, the *E*_*loc*_ intensity in the central area of CPP subjects is also lower than that of the other two groups, and the overall *E*_*loc*_ intensity of DD subjects is lower than that of the other two groups. In the distribution of *CC*, the overall distribution of the three groups is similar, but the left frontal lobe intensity is higher in the CPP and DD groups.

### 3.3. Classification accuracy of different frequency bands

**FIGURE 8:**
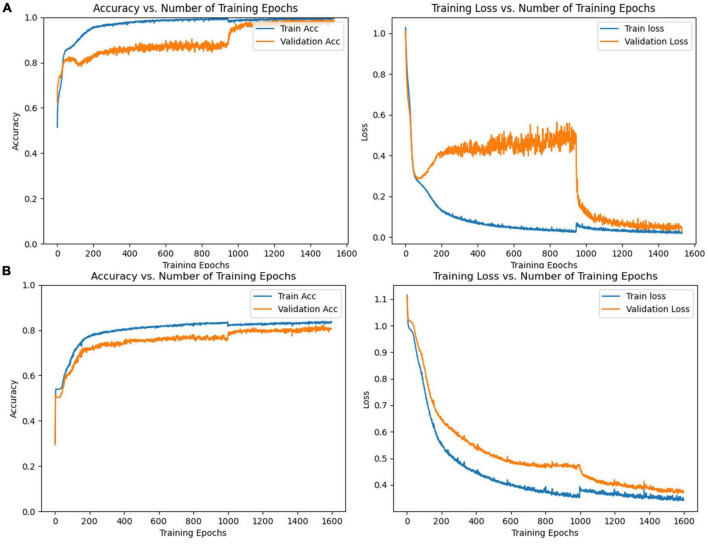
**(A)** Accuracy and loss rate change curve of training set and validation set trained with EEG, **(B)** accuracy and loss rate change curve of the training set and validation set trained with PLI matrix.

**FIGURE 9:**
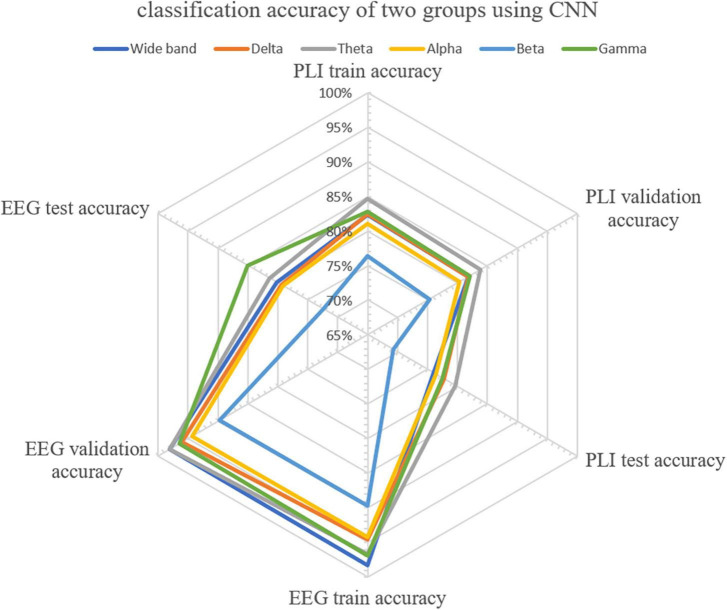
Accuracy distribution of classification of EEG and PLI matrices of three groups of people using CNN in six frequency bands.

**FIGURE 10:**
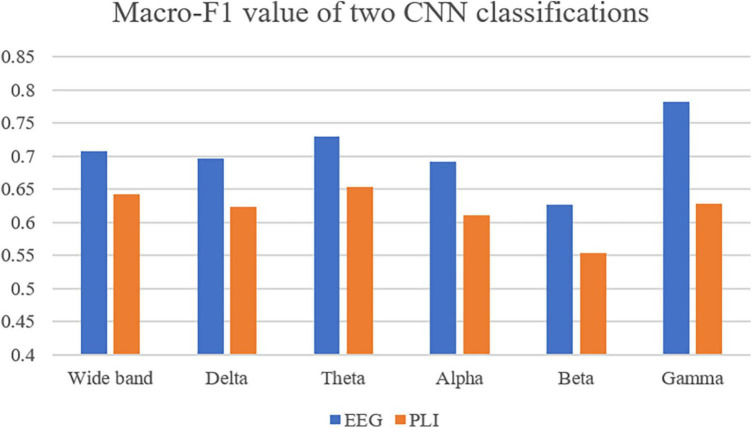
Use Macro-F1 to evaluate the model obtained from two kinds of data in six frequency bands.

CNN was first used to classify the EEG of three groups of subjects. Further, this paper used the improved CNN model to classify the PLI matrix of three groups of subjects to verify the above brain network analysis results. After 1,000 training epochs, the loss rate, and verification loss rate of the two classifications began to converge to 0 and 0.8, respectively, while the training accuracy and verification accuracy converged to 1 and 0.3, respectively, which proved that the network structure was stable (Figure 8). Figure 9 shows that both sets of classifications have achieved ideal accuracy in six frequency bands: in the classification of EEG, the Gamma band has the highest accuracy of 85.01%, followed by the Theta band with 81.37%; in the classification of PLI, the highest frequency band of Theta is 79.64%, followed by Delta and Gamma with 77.81 and 77.47%. The results of Macro-F1 in Figure 10 show that the classification model of EEG is better than that of PLI. Except for the Beta frequency band, the Macro-F1 evaluation of other frequency bands are above 0.65, and the PLI classification model is also above 0.6 except for the Beta frequency band.

## 4. Discussion

In this study, the CNN algorithm was used to classify CPP, DD, and HC through EEG activities evoked by the auditory oddball paradigm, and the brain network and complex connectivity of the three groups were investigated. Our main results show that the deep learning approach of CNN exhibits successful classification for all the three groups, with the highest classification accuracy in the Gamma band and the second highest classification accuracy in the Theta band. Brain network analysis reveals a stronger phase-based connection within the frontoparietal network in the Theta band in CPP subjects. The same trend is also reflected in the degree centrality and global efficiency.

The analysis of the characteristics of CPP brain network connectivity in this study reveals that the connectivity of the brain networks in the prefrontal and posterior area of CPP patients is significantly increased in the Theta band. Graph theory analysis shows that CPP patients has increased the overall efficiency and stronger centrality at the node level in the right frontal regions and left parietal occipital regions. Despite the limited spatial resolution of the EEG, it has been found that the area with increased network connectivity in the Theta band characteristic of CPP is highly overlapped with frontoparietal network (FPN). This network is also often referred as the central executive network, whose activity is important for goal-directed cognitive tasks, including working memory, planning, judgment, and decision-making (Kelly et al., 2008; Menon, 2011). Important FPN regions are the dorsolateral prefrontal cortex and the posterior parietal cortex. Previous studies have shown that the increased functional connectivity in the prefrontal cortex is crucial for the chronicity of pain (Baliki et al., 2012; Hashmi et al., 2013; Vachon-Presseau et al., 2016). At the same time, the function of prefrontal cortex is closely related to the perception of chronic pain and its related negative emotions, cognitive changes, and avoidance behaviors (Baliki and Apkarian, 2015; Kragel et al., 2018; May et al., 2019). Theta event-related synchronization (ERS) observed in prefrontal regions during the oddball task is thought to primarily reflect activation of neural networks involved in allocation of attention related to target stimuli (Missonnier et al., 2006). Migraine patients presented an enhanced phase-synchronization in the theta frequency range during auditory attention tasks (Vilà-Balló et al., 2021). Similar to our results, these findings could indicate the presence of a hypersensitivity to auditory stimuli and hyperexcitability in attentional control in CPP patients. Therefore, the neurological profile of CPP patients may be characterized by impaired FPN function, resulting in dysfunctional adaptive strategies for individuals to perceive stimulus (including pain) both cognitively (how the individual feels) and behaviorally (what the individual does).

EEG has been used to classify depression correctly with great accuracy in many studies (de Aguiar Neto and Rosa, 2019). Many previous studies analyzed the oscillatory power spectrum at rest. We first investigate neural response induced by oddball task in CPP and DD patients. Our deep learning results show that the classification accuracy of EEG activities in the theta, alpha, beta and gamma bands can reach more than 80% during the oddball task for the three groups, with the best classification accuracy in the gamma band. Furthermore, the classification model of EEG is better than that of connectivity (i.e., PLI). Neural activity evoked by deviant stimuli in oddball paradigm has been related to attention and memory updating for discrete events. A significant increase in the relative power of theta can be observed in healthy subjects, while a decrease in the higher frequency bands (Gomez-Pilar et al., 2016). Theta oscillatory response induced by oddball paradigm is restricted to the bilateral frontal cortex, particularly in the dorsolateral, and medial prefrontal areas, while 30–60 Hz bands can be visualized over the bilateral central region (Ishii et al., 2009). Theta oscillation is considered to be closely related to cognitive functions, such as attention and memory processing (Buzsáki, 2002; Wang and Ding, 2011). Gamma oscillation may represent the basic characteristics of neuron signal and communication, which seems to be particularly relevant to the local processing and feedforward communication of current important stimuli (Donner and Siegel, 2011; Fries, 2015; Ploner et al., 2017). Interestingly, resting-state Gamma in prefrontal cortex, on one hand, has been considered as a reliable marker for major depression, as this brain region is heavily implicated in mood and emotional regulation (Fitzgerald and Watson, 2018). On the other hand, a latest systematic review reports Gamma oscillations in the prefrontal cortex as a promising biomarker for tonic and chronic pains (Li et al., 2023). Considering resting-state activities could impact task-induced activities, the results we found for optimal classification accuracy in the gamma band may be interpretable.

Our findings reveal that significant difference in brain network connectivity (i.e., PLI) is in Theta band, while the second highest accuracy in EEG classification is achieved in the Theta band, and the highest classification accuracy is in the Gamma band. This could be explained by following reasons. First, the classification accuracy of PLI matrix is generally lower than that of EEG. This difference is due to the loss of characteristic information such as the time domain and frequency domains when using PLI to calculate brain network connection matrix. Second, it has been observed that there is significant phase synchronization between frontal and posterior electrodes during auditory oddball task in the Gamma and Theta bands, which has been interpreted as a functional connectivity among cortical regions devoted to the task execution (Choi et al., 2010). Given the vital role of Gamma activity in the neural communications between different brain structures and networks (Fries, 2015), our result could be related to this Theta-Gamma synchronization. Future analysis on Theta-Gamma synchronization may further explore the underlying neural mechanisms.

The current deep learning method shows that it is of great significance to apply CNN to EEG data of auditory stimulation state to distinguish among CPP, DD, and HC subjects with an accuracy rate of more than 80%. First, it suggests that the EEG of each frequency band may play a role in the pathophysiology of CPP. Second, the current approach may be a step toward an EEG-based auxiliary diagnosis of CPP. Third, the abnormal pattern of EEG activity in CPP patients may represent a potential new therapeutic target, such as intervention through non-invasive brain stimulation technology or neural feedback methods. In particular, the emerging transcranial magnetic stimulation can modulate the oscillation and synchronization of neurons at specific frequencies, so it may be a promising method for pain modulation (Polanía et al., 2018).

## 5. Conclusion

In conclusion, our results indicate that there are significant differences among CPP, DD, and HC subjects in the characteristics of auditory oddball-induced EEG activities, suggesting that CPP has its unique neuroelectrophysiological manifestations. The connectivity of theta band in FPN-related brain regions is significantly enhanced in CPP patients, thus contributing to better understanding of the brain mechanism of CPP. Our research provides a novel approach for the objective assessment of CPP. The non-invasive brain stimulation and neural feedback approaches targeting Theta and Gamma oscillation and FPN networks may be a potential treatment scheme.

## Data availability statement

The raw data supporting the conclusions of this article will be made available by the authors, without undue reservation.

## Ethics statement

The studies involving human participants were reviewed and approved by the experimental procedures involving human subjects described in this paper were approved by the Shanghai Changhai Hospital Review Board. The patients/participants provided their written informed consent to participate in this study.

## Author contributions

YL: software, validation, writing – original draft, and visualization. BY: conceptualization, methodology, resources, project administration, and funding acquisition. ZW: resources, data curation, and project administration. RH and XL: data curation. XB: conceptualization, investigation, and project administration. SZ: methodology, validation, writing – review and editing, and funding acquisition. All authors contributed to the article and approved the submitted version.
