# Supplementary material for: EEG assessment of brain dysfunction for patients with chronic primary pain and depression under auditory oddball task
Source: Front Neurosci. 2023 Mar 24;17:1133834. doi: 10.3389/fnins.2023.1133834 (PMC10079993; doi:10.3389/fnins.2023.1133834)
Supplement: Supplementary file 1 [file Data_Sheet_1.docx]

Supplementary Material

EEG Assessment of Brain Dysfunction for Patients with Chronic Primary Pain and Depression under Auditory Oddball Task

Yunzhe Li, Banghua Yang^*^, Zuowei Wang, Ruyan Huang, Xi Lu, Xiaoying Bi^*^, Shu Zhou^*^

*** Correspondence:**
Banghua Yang, Xiaoying Bi, Shu Zhou
yangbanghua@shu.edu.cn, bixiaoying2013@163.com, zhoushu.49@hotmail.com

# Supplementary Figures and Tables

## Supplementary Figures


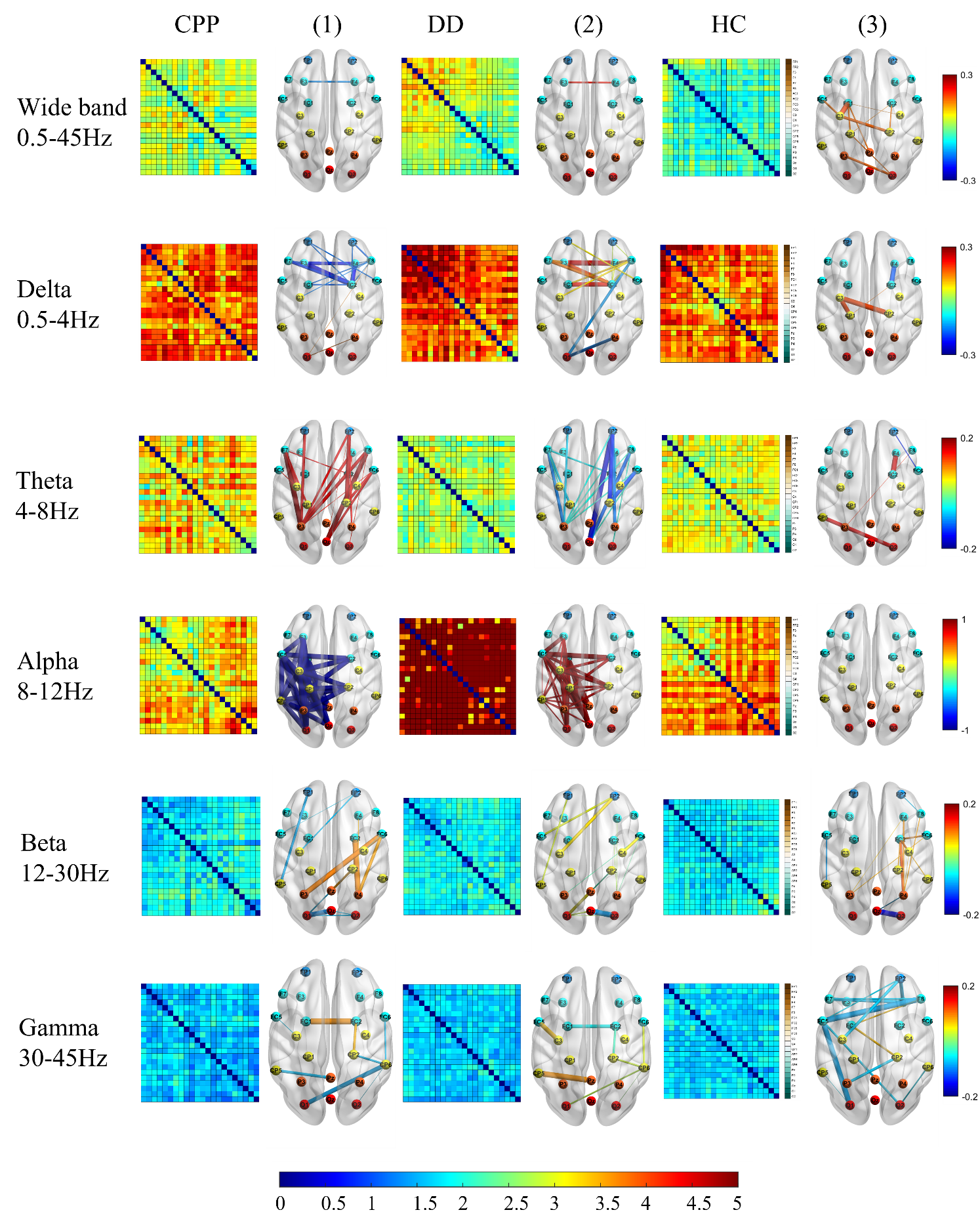


**Supplementary Figure 1.** This figure shows the value range of the functional connection matrix sum of CPP, DD and HC in each frequency band is [0,5], and its electrode channels are FP1, FP2, F3, F4, F7, F8, FC1, FC2, FC5, FC6, C3, C4, CP1, CP2, CP5, CP6, Pz, P3, P4, Oz, O1, O2 from top to bottom. (1) (2) (3) The figure shows that subtracting matrix values (CPP-DD, DD-HC, CPP-HC, respectively), the most significant difference feature remains 30%. The value range is shown on the far right. The Wide band is [- 0.3, 0.3], Delta is [- 0.3, 0.3], Theta is [- 0.2, 0.2], Alpha is [- 1, 1], Beta is [- 0.2, 0.2], and Gamma is [- 0.2, 0.2].


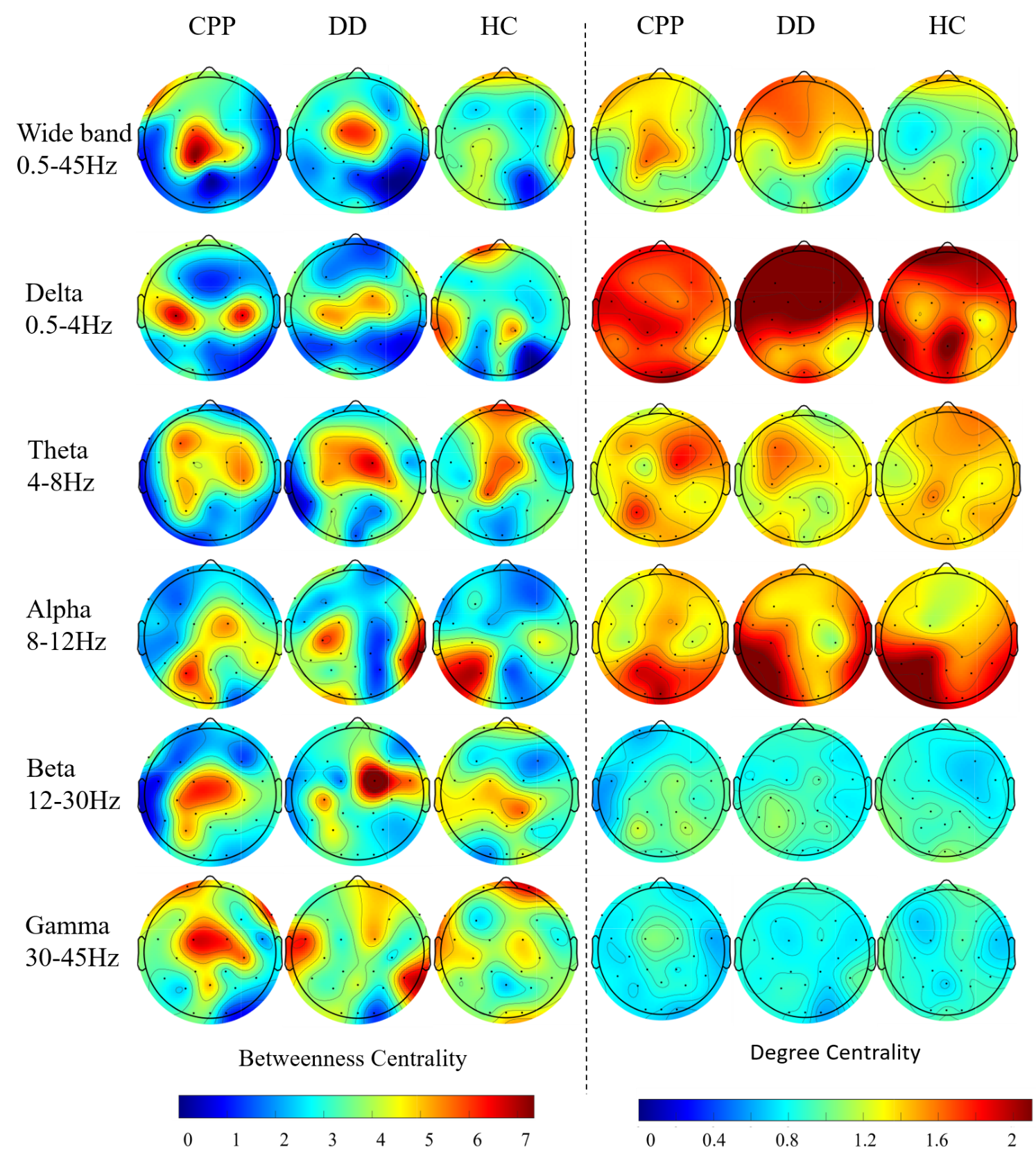


Supplementary Figure 2. The left figure shows the average betweenness centrality distribution of CPP, DD and HC in each frequency band, with a value range of [0,7]. The right figure shows the average distribution of CPP, DD and HC in each frequency band, with a value range of [0,2].


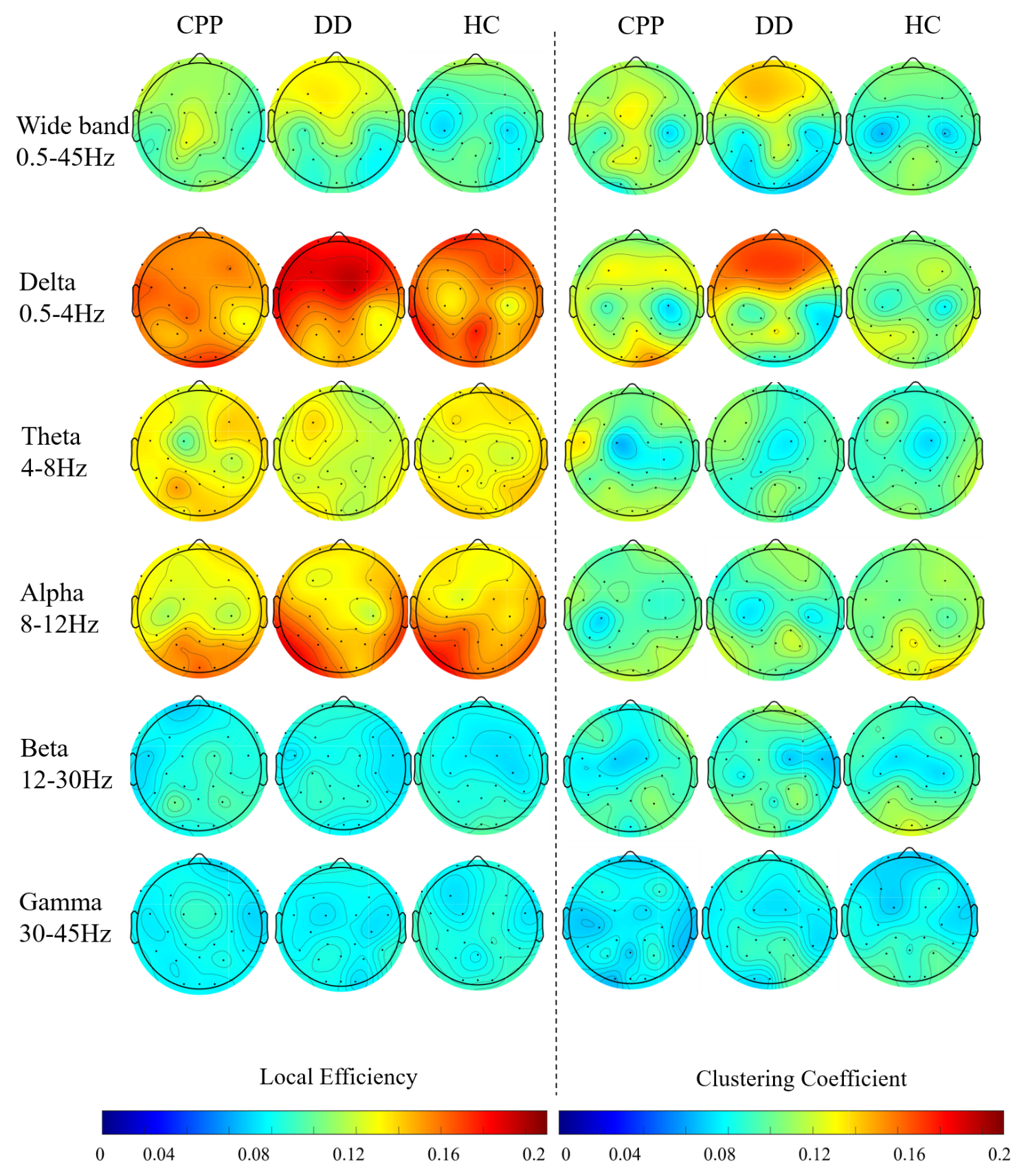


Supplementary Figure 3. The left figure shows the average local efficiency distribution of CPP, DD and HC in each frequency band, with a value range of [0,0.2]. The right figure shows the average distribution of CPP, DD and HC in each frequency band, with a value range of [0,0.2].
